# First Description of a Primitive Neuroectodermal Tumor Arising in the Nose

**DOI:** 10.1155/2013/512416

**Published:** 2013-05-22

**Authors:** Habib Rizk, Aline Khazzaka, Amer Sebaaly, Maguy Cherfan, Roland Tomb, Riad Sarkis

**Affiliations:** ^1^Department of Otolaryngology Head and Neck Surgery, Hotel Dieu de France Hospital, Beirut, Lebanon; ^2^Surgical Research Laboratory, Saint Joseph University Medical School, Medical Sciences Campus, B.P. 11-5076, Riad El Solh, Beirut 1107 2180, Lebanon; ^3^Department of Orthopedic Surgery, Hotel Dieu de France University Hospital, Beirut, Lebanon; ^4^Pathology Department, National Institute of Pathology, Beirut, Lebanon; ^5^Department of Dermatology, Hotel Dieu de France Hospital, Beirut, Lebanon; ^6^Department of General Surgery, Hotel Dieu de France Hospital, Beirut, Lebanon

## Abstract

We report the case of a 12-year-old girl, who consulted us with one-year history of an 8 mm nose lesion that was painless and firm upon palpation. The lesion was resected conservatively. Immunohistochemistry was in favor of a primitive neuroectodermal tumor (PNET)/Ewing's sarcoma lesion, excluding epithelial, lymphoid, and other tumors. After a second resection, our patient was referred to chemotherapy and has already undergone 9 cycles out of 14. The patient is to date with no evidence of persistent or recurrent disease. To our knowledge, this is the first description of a PNET arising in the nose.

## 1. Introduction

Primitive neuroectodermal tumors (PNETs) are a large family of tumors of neuroectodermal origin that belong to Ewing's sarcoma family of tumors [1–4]. Head and neck localizations of these tumors are rare especially in children [3, 5, 6]. The most common location in the head and neck region is the orbit followed by the neck and the parotid gland [2, 3]. Because of the aggressive nature and poor prognosis, meticulous surgical and medical treatment regimens are needed. We herein report the first case in the literature of PNET arising in the nose and review the morphologic and immunohistochemical findings of this tumor with a review of the literature.

## 2. Case Report

**Figure 1:**
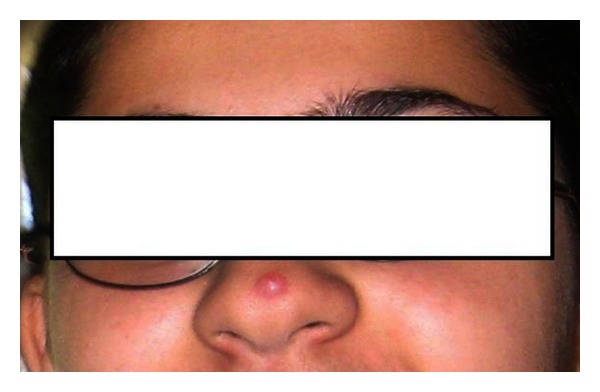
An 8 mm firm, nonpainful nose lesion increasing in size over the span of one year.

The authors report here the case of a 12-year-old girl that presented with tip of nose lesion a year ago. This lesion was asymptomatic and barely increased in size in the first 3 months. She presented to our clinic in September 2011 with an 8 mm lesion that was painless and firm upon palpation (Figure 1). This lesion was resected conservatively and sent to pathology. 

Tissue from the tumor was processed routinely for light microscopy. Immunohistochemistry was performed in paraffin-embedded tissue using commercially available antibodies against *leukocyte common antigen (LCA), cytokeratin cocktail (CKC), synaptophysin, chromogranin, S-100, CD99, myogenic differentiation 1 (MyoD1)*, *glial fibrillary acid protein (GFAP), neuron-specific enolase,* and *Ki-67* according to a standard immunohistochemical protocol. 

Histology revealed a neoplastic malignant polypoid proliferation, located in the lamina propria under an intact stratified squamous epithelium, composed of sheets of small, round to oval blue cells, arranged in lobules and separated by fibrous septa. Cells were characterized by scant, ill-defined cytoplasm and well-defined nuclei with coarse chromatin pattern. There was no rosettes formation. No areas of necrosis were noted, neither signs of differentiation toward heterologous elements. 

**Figure 2:**
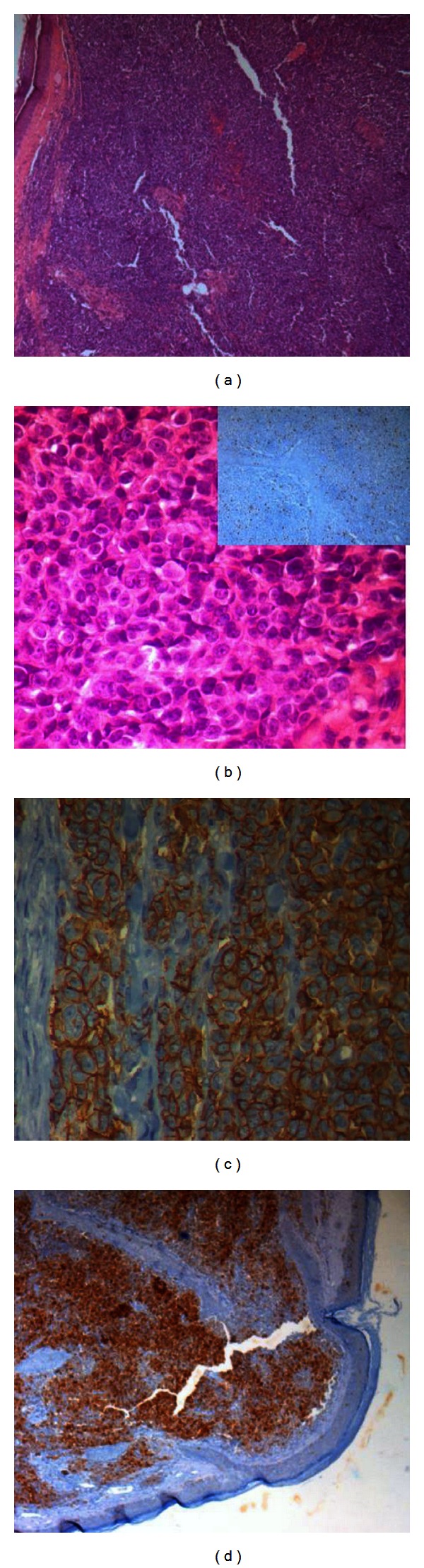
(a) Lobules of small, round, blue cells, separated by fibrous septa underneath an intact squamous epithelium (hematoxylin-eosin stain, objective original magnification ×5). (b) Higher magnification revealing tumor cells with scant, ill-defined cytoplasm and well-defined nuclei with coarse chromatin pattern (objective original magnification ×40), with a proliferation index superior to 15% (Ki-67 stain at the upper right corner). (c) Immunohistochemical membranous cytoplasmic stain for CD99 (objective original magnification ×40). (d) Immunohistochemical stain for S-100 (objective original magnification ×5).

**Figure 3:**
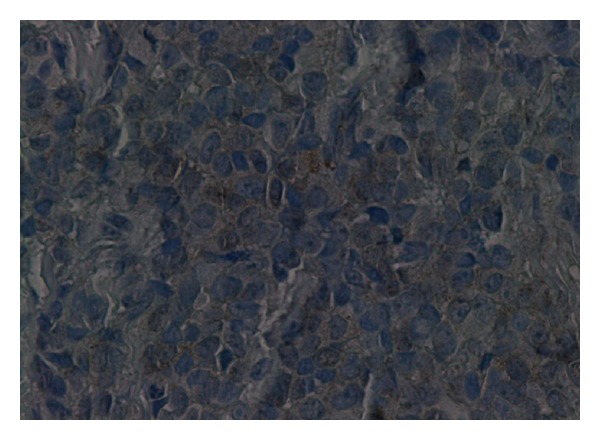
Neuron-specific enolase stain showing mild positivity.

The immunohistochemistry study showed strong positivity for *CD 99, S-100,* and *neuron-specific enolase* and negativity for the other markers, with a proliferation index superior to 15% in favor of a PNET/Ewing's sarcoma lesion, excluding epithelial, lymphoid, and other tumors (Figures 2 and 3).

Given the pathology result, a second look with resection to ensure safe margins was done. The patient was then referred to chemotherapy. She is currently in her 9th cycle out of 14 cycles. The protocol consists of alternated cycles of vincristine, doxorubicin, and cyclophosphamide and alternate cycles of ifosfamide and etoposide. Doxorubicin is withheld in cycles 11 and 13.

The patient is to date with no evidence of persistent or recurrent disease.

## 3. Discussion

Peripheral PNET encompasses a large spectrum of neoplasms ranging from the least differentiated Ewing's sarcoma to the most differentiated peripheral neuroepithelioma including Askin's tumor, melanotic neuroectodermal tumor, ectomesenchymoma, and peripheral medulloepithelioma [7]. The unifying term Ewing's sarcoma family of tumor encompasses these tumors because of similar histological and immunohistochemical characteristics as well as cytogenetics [8]. Pathologically, they might represent a transition between neoplastic Schwann cells, neuroblasts, and perhaps paraganglionnic elements [8].

Malignant peripheral PNET neuroepithelioma represents less than 1% of all sarcomas. It usually presents as a rapidly enlarging painful mass. Approximately one third of cases are attached to a major peripheral nerve [2, 3].

Head and neck localizations of these tumors are rare. However, it is noteworthy that one case series by Jones and McGill found that head and neck localizations came second after thoracopulmonary involvement [8]. These tumors present mostly in thoracopulmonary region, the abdomen, and the extremities. In the head and neck the most common location is the orbit followed by the neck and the parotid gland [2, 3]. 

Diagnosis relies mainly on histological, immunohistochemical, and ultrastructural findings. Neural differentiation is usually indicated by the presence of rosettes of the Homer-Wright type. Immunohistochemically, MIC2 (CD99) is usually present as well as other neural differentiation markers such as neuron-specific enolase, S-100, synaptophysin, and chromogranin [2–4]. Although MIC2 reactivity is very sensitive, it is not highly specific as it can be found in T-cell lymphoblastic leukemia, certain lymphomas, synovial sarcomas, Wilm's tumor, rhabdomyosarcoma, small cell osteosarcoma, and intraabdominal small round cell tumor [4]. Ultrastructural features show neurosecretory granules. Finally, cytogenetic studies can look for the characteristic translocation t (11; 22) (q 24;12) that causes mutation of EWS gene resulting in EWS-FLI1 fusion protein identified in 85%–90% of peripheral PNET neuroepithelioma and Ewing's sarcoma [2, 4, 8].

There are no characteristic radiologic presentations but peripheral PNET (pPNET) presents as a heterogeneous mass on CT scan, isointense to muscle on T1-weighted MRI but with homogeneous enhancement with gadolinium and nonhomogeneous hyperintensity on T2-weighted MRI [9–11]. These tumors usually exhibit central necrosis and vessel invasion and rarely exhibit intralesional calcifications [9].

The most difficult task is to differentiate pPNET from Ewing's sarcoma, and this diagnosis is made on the basis of neural differentiation since both entities represent a spectrum of the same disease [2, 8].

Combination treatment modalities are used in this type of tumor, most frequently surgery followed by radiation for local control and chemotherapy. Inoperable tumors are treated with concomitant chemoradiation protocols [3–5]. Recommendation for radiation depends on primary site and size of tumor, histology, patient's age, and extent of tumor before and after surgical resection [3–5]. Sometimes chemotherapy is used preoperatively followed by surgery especially when initial resection would be mutilating because of the proximity of vital structures. Chemotherapy protocol advocated is a high-risk sarcoma protocol [2, 3]. Thus, dactinomycin, vincristine, alkylating agents such as cyclos, phophamide and ifosfamide and anthracyclines such as doxorubicin (adriamycin) and epidoxorubicin have proved to be useful in PNET [3].

Prognosis is usually poor with less than 50% of disease-free survival at three years and 30%–45% at 5 years [5]. These tumors have a propensity to early distant metastasis to lung, liver, and bone marrow. Prognosis is dictated by negative margins at first excision and also by primary tumor location. Head and neck tumors had an intermediate outcome with tumors arising in paraspinal and scapular areas with the most favorable outcome and abdominal tumors unresponsive to treatment. Finally, the orbital location seems to be associated with a particularly better prognosis too [2, 4, 5]. Poor prognostic factors for PNETs include age superior to 10 years, bony infiltration and bone marrow metastasis, radiation therapy at doses greater than 55 Gy, and high-dose chemotherapy with hematopoietic stem cell rescue [12]. Due to the rarity and biological aggressiveness of PNET, multi-institutional clinical trials are necessary to formulate more effective therapeutic regimen [12].
